# Real-world data on triple-negative breast cancer in Latin America and the Caribbean

**DOI:** 10.3332/ecancer.2023.1635

**Published:** 2023-11-21

**Authors:** Katsuki Arima Tiscoski, Juliana Giacomazzi, Matheus Soares Rocha, Gustavo Gössling, Gustavo Werutsky

**Affiliations:** 1 Santa Casa de Misericórdia de Porto Alegre, Rua Professor Annes Dias, Porto Alegre 90020-090, Brazil; 2 Latin American Cooperative Oncology Group (LACOG), Av Ipiranga, Porto Alegre 90619-900, Brazil; a https://orcid.org/0000-0003-0074-4272; b https://orcid.org/0000-0001-5811-5140; c https://orcid.org/0000-0001-8972-7449; d https://orcid.org/0000-0002-4361-2889; e https://orcid.org/0000-0001-6271-105X

**Keywords:** breast neoplasms, Latin America, Caribbean Region, triple negative breast neoplasms

## Abstract

Breast cancer (BC) is the most prevalent cancer in women in Latin America and the Caribbean. We compiled real-world data (RWD) on the epidemiology, diagnosis, treatment, and patient outcomes of triple-negative breast cancer (TNBC), addressing the main barriers to optimal care in Latin America. The prevalence of TNBC varies between 11% and 38.5% of all BC cases diagnosed in the region, and TNBC primarily affects young patients. Delays in BC diagnosis, with consequent advanced disease stages and barriers to access efficient therapies, particularly due to high costs, negatively impact patient outcomes. Cancer clinical trials are an opportunity to access standard and novel therapies for patients with this aggressive BC subtype and thus must be prioritised. Finally, generating RWD and cost-effectiveness studies in a region with limited resources is critical for decision-makers to define the incorporation of new technologies for the treatment of BC.

## Introduction

Breast cancer (BC) is the most common cancer among women worldwide, and according to GLOBOCAN, in 2020, the incidence was 2,261,419 new cases and 684,886 deaths [1]. In Latin America and the Caribbean (LAC), BC was responsible for 14% (210,000 new cases) of all cancer cases in 2020, with 57,984 deaths, and it’s estimated that BC will increase to approximately 314,000 new cases per year and 94,600 deaths by 2040 [2].

Inequities in access to cancer care in LAC countries translate into unequal outcomes [3]. However, it is difficult to measure because of the absence of cancer registries and low data quality [4–6]. In Latin America, cancer registries cover only 7%–8% of the population, while the equivalent coverage is 83% in North America and 32% in Europe [7, 8]. Epidemiological and clinical data on BC subtypes are available in only a few observational studies in the region; thus, it is a challenge to evaluate a particular BC subtype, such as triple-negative breast cancer (TNBC).

Among all invasive BC, 10%–17% are classified as TNBC, an aggressive disease subtype that affects a considerable number of Latin American women. TNBC generally occurs in young patients, particularly in those under 40 years of age, is frequently diagnosed in locally advanced stages, and is associated with *BRCA* mutations. TNBC has a poor prognosis with approximately 30%–40% disease recurrence, often involving visceral organs. Patients with metastatic disease have a survival time of approximately 15 months [9–14]. Although new drugs that significantly impact TNBC outcomes in early and metastatic stages have been approved in recent years, limited access exists for most patients in LAC countries. Therefore, we aimed to compile real-world data (RWD) on TNBC to describe its epidemiology, diagnosis, treatment access, and patient outcomes in LAC.

## Methods

To explore the RWD about TNBC in LAC, an advanced literature search was performed through the PubMed database using the following strategy: ((((((((((triple negative) OR (triple-negative)) OR (triple negative[MeSH Terms])) OR (breast cancer[MeSH Terms]))) AND (((breast cancer) OR (breast cancer[MeSH Terms]))))) AND (((((latin america) OR (latin america[MeSH Terms]))) AND (((caribbean) OR (caribbean[MeSH Terms]))))))) AND (((((((((((((((((((((Brazil) OR (argentina)) OR (mexico)) OR (chile)) OR (peru)) OR (colombia)) OR (guatemala)) OR (panama)) OR (costa rica)) OR (venezuela)) OR (cuba)) OR (ecuador)) OR (uruguay)) OR (el salvador)) OR (honduras)) OR (dominican republic)) OR (bolivia)) OR (nicaragua)) OR (paraguay)) OR (haiti)))). Gray literature was also accessed to search for scientific publications on TNBC in LAC. Sixty-five manuscripts have been revised.

Additionally, a survey was administered to oncologists from countries affiliated with the LACOG to demonstrate the availability of novel therapies for treating TNBC from public and private coverage in LAC countries.

A search in the National Library of Medicine/Clinicaltrials.gov was performed to detail the ongoing clinical research in LAC, and the following keywords and criteria were used: ‘oncology’ in the ‘condition or disease’ field; ‘triple-negative’ in the ‘other terms’ field. It was selected in the status field – ‘recruiting’, ‘not yet recruiting’, ‘active’, ‘not recruiting’, ‘completed’, ‘enrolling by invitation’,; ‘study type’ field – ‘interventional studies’; and ‘phase’ field – ‘early phase 1’, ‘phase 1’, ‘phase 2’, ‘phase 3’, ‘phase 4’. The final date in the ‘study start’ field was February 23, 2023.

### Epidemiology of TNBC in LAC

Several epidemiological studies have reported a higher prevalence of TNBC in Latin American women than in non-Hispanic women [15–18]. Table 1 shows the prevalence of TNBC reported in studies conducted in 11 LAC countries. The frequency of TNBC ranges between 11% and 38.5%, with the highest rates reported in Peru and Haiti.

Studies have shown that TNBC is more prevalent in young women. In a prospective cohort study from Brazil with more than 3,000 BC patients, those aged <40 years were more frequently diagnosed with TNBC.

Furthermore, 20% of deaths due to BC at a younger age are caused by this subgroup, which differs from that in developed countries, where the deaths correspond to less than 12% and 10%, respectively [19, 20].

A study conducted in Peru including 1,582 adolescents and young adult females with BC demonstrated that although adolescents and young adult females have more aggressive clinical features at diagnosis, survival outcomes were comparable with those of middle-aged and older women with TNBC (5-year overall survival/event-free survival for adolescents and young adults was 55%/53%, similar to middle-aged (54%/49%) and older females (56%/51%). This suggests that age is not a risk factor for worse survival outcomes if treatment is administered according to cancer stage [21].

### Pathology and genetic testing

Molecular testing and drug access also vary significantly across LATAM countries. Few studies have investigated the level of discordance and the quality aspects of ER/PgR and Human Epidermal Growth Factor Receptor 2 (HER2) immunohistochemistry tests in LAC. For example, a study examined the concordance in the results of HER immunohistochemistry assays performed on 500 invasive breast carcinomas between a reference laboratory and 149 local laboratories from all geographic regions of Brazil. The results showed an overall poor concordance of 34.2% regarding HER2 results between local and reference laboratories [36]. fluorescence *in situ* hybridisation or chromogenic *in situ* hybridisation techniques are only available in highly specialised laboratories and institutes, and the outsourcing of this service increases the delay in the diagnosis of BC [37]. Analysis of cancer-specific markers, such as PD-L1, required for the administration of immunotherapy in advanced disease, when available, is offered only for patients with private insurance or through programs provided by the pharmaceutical industry. However, limited access to tests of cancer-specific markers remains in countries without a commercial supplier [38, 39].

The capacity for and development of cancer genomics in Latin America was described in a recent study. It identified 221 next-generation platforms currently available in the region. Mexico, Brazil, Chile, Argentina, and Colombia are the leading countries in installed facilities, cancer genetics research groups, educational programs in genomics, and medium-impact publications in the field. Meanwhile, countries in Central America were shown to be underrepresented in all areas of ongoing cancer genomic development and implementation [40]. These disparities impact genomic testing and analysis in different clinical scenarios, such as cancer prevention (identification of high-risk cancer genes), tumour genomic profiling for diagnosis and prognosis, and personalised treatment [40–44].

In Brazil, in a subanalysis of the AMAZONA observational study including 2,950 patients, 1,094 (37%) had at least one criterion for hereditary breast and ovarian cancer syndrome. Of all patients, only 45 (6.9%) underwent BRCA testing, and of those tested, 18 (40%) had an identified pathogenic mutation [45]. Among Latin American cancer patients, the frequency of pathogenic variants in the BRCA gene has been reported to be between 1.2% and 15.6% [46–49] and between 15% and 28% of breast and ovarian cancer patients unselected for family history of BC in Mexico [50].

For BRCA1/2 testing, LAC laboratories use state-of-the-art platforms with similar quality control metrics and variant classification protocols as laboratories in Europe and other areas of the world [51]. Quality standards for pathology tests (e.g., immunohistochemistry) are still a matter of concern in LAC, and access to genetic testing is far from ideal because of its high cost and lack of insurance coverage for supportive healthcare policies [40, 44, 52].

### Treatment and outcomes of TNBC

The treatment of TNBC has improved in recent years with the incorporation of new therapies for both early and advanced disease. However, the delay in diagnosis and initiating adjuvant systemic treatment is commonly described in countries from LAC, and it's associated with worsening clinical outcomes [22, 32, 53–64]. For example, Morante *et al* [65] showed that the 10-year-overall survival of patients with TNBC who started chemotherapy ≤ 30 days after surgery was 82% versus 65.1% for those patients who started treatment after ≥ 91 days [65].

Novel agents approved for treating metastatic TNBC, such as Poly ADP-ribose polymerase (PARP) inhibitors, immunotherapy, and antibody-drug conjugates, are not widely accessible to patients in LAC, and thus most patients from the public health system in LAC are still exposed to conventional chemotherapy only. The scenario of access to TNBC therapies by public and private health systems in eight LAC countries was evaluated through a survey conducted by oncologists working in these countries (Table 2).

At the time of our survey (May 2023), pembrolizumab in the neoadjuvant setting was not available in any country in the public health system. In the metastatic setting, pembrolizumab was available in the public health system in only two countries (Argentina and Colombia). PARP inhibitor was available in Argentina, Colombia, and Costa Rica in the public health system. In the private health system, pembrolizumab in the neoadjuvant setting is available in all countries except Colombia and Uruguay, the latter also being the only country where pembrolizumab is not available in the metastatic setting. Sacituzumab is available only in Brazil, and PARP inhibitors for metastatic disease are available in all countries except Uruguay (Table 2).

### Clinical trials in TNBC

In the last 5 years, 19 (5.8%) of 323 TNBC clinical trials involving nine LAC countries were registered in the National Library of Medicine (www.clinicaltrials.gov) [66]. Among them, 16 (84.2%) were not recruiting or active. Of the total trials in LAC, 3 (15.8%) were phase I trials, 3 (15.8%) were phase 2 trials, and 13 (68.4%) were phase 3 trials (Table 3).

The countries most cited as participating sites in clinical trials were Mexico (12 trials; 20.3%), Brazil (11 trials; 18.6%), Argentina (11 trials; 18.6%), Chile (8 trials; 13.6%), and Peru (7 trials, 11.9%). Colombia, Costa Rica, Panama, and Cuba represented less than 15% of the trials in the LAC. No clinical trials have been registered in Guatemala, Venezuela, Ecuador, Uruguay, El Salvador, Honduras, Dominican Republic, Bolívia, Nicaragua, Paraguay, or Haiti. The industry sponsors an average of 85.7% of the TNBC clinical trials (Table 3). The drug categories evaluated in these studies were inhibitor checkpoint (*n* = 8; 42%), conjugated antibodies (*n* = 4; 21%), inhibitor AKT Kinase (*n* = 3; 16%), vaccine (*n* = 2; 11%), PARP inhibitors (*n* = 1; 5%), and inhibitor PIK3CA (*n* = 1; 5%) (Table 3).

## Conclusion

The prevalence of TNBC in LAC varies between 11% and 38.5% of all BC cases diagnosed in the region and affects mostly young patients. Delays in BC diagnosis, barriers to pathology, and genetic testing affect patient outcomes.

Novel drugs that significantly affect survival have been incorporated into the private health system in the majority of LAC countries. Nonetheless, as more than 80% of the LAC population is covered by the public health system, chemotherapy is the only systemic treatment available. Generating RWD and cost-effectiveness studies on LAC is critical for deciding the incorporation of new technologies considering the country's limited resources.

## Conflicts of interest

JG reports grants from Roche. GW reports grants or contracts from Novartis, Roche/Genentech, AstraZeneca/MedImmune, Lilly, GlaxoSmithKline, Novartis, Pfizer, Bristol-Myers Squibb Brazil, MSD, Merck, Bayer, Janssen, BMS, Astellas, Libbs, Takeda, Celgene, GSK; consulting fees from Merck; payment or honoraria for lectures from Pfizer, AstraZeneca/MedImmune, Libbs, and Merck. The other authors declare no conflict of interest.

## Funding

This study received no funding.

## Ethical statement

No ethical approval was required for this review paper, as it does not involve primary research on human subjects, animal experimentation, or the collection of personally identifiable information. We adhered to ethical guidelines for proper citation, referencing, and avoidance of plagiarism, ensuring the appropriate attribution of sources, and upholding ethical standards in our research and publication practices.

## Author contributions

In accordance with the guidelines set forth by the International Committee of Medical Journal Editors (ICMJE), all authors of this paper made substantial contributions to the conception, design, execution, and interpretation of the research study. All authors have read and approved the final version of the manuscript and take full responsibility for its content.

## Figures and Tables

**Table 1.** Prevalence of TNBC in LAC countries.

Country	Triple-negative (%)	Reference	Source
Haiti	38.5	DeGennaro *et al* [22]	Innovating Health International (Research Institute)
Mexico	16–23.1	Lara-Medina *et al* [23], Valdez *et al* [24]	Cancer Institute
Costa Rica	17.1–22.22	Srur-Rivero and Cartin-Brenes [25], Quirós-Alpízar *et al* [26]	Hospitals-based
Peru	21.3–30.8	Vallejos *et al* [27], Mendoza-del Solar and Cervantes-Pacheco [28]	Hospitals-based
Colombia	20.6	Serrano-Gomez *et al* [29]	National Cancer Institute of Colombia
Guatemala and Honduras^a^	18.12	Reyes-Morales *et al* [30]	Hospitals-based
Uruguay	16.0	Delgado *et al* [31]	Hospitals
Brazil	15.6	Rosa *et al* [32]	Hospitals-based
Argentina	15.3	de Almeida *et al* [33]	Hospitals-based
Chile	11–14.8	Walbaum *et al* [34], de Almeida *et al* [33], Acevedo *et al* [35]	Hospitals-based

a Grouped due to overlap of data

**Table 2.** Access to novel therapies for the treatment of BC in LAC.

Country	Scenario/Medication							
	Neoadjuvant/Pembrolizumab		Metastatic/Pembrolizumab		Sacituzumab		PARP inhibitor	
	Public	Private	Public	Private	Public	Private	Public	Private
Argentina	-	✓	✓	✓	-	-	✓	✓
Bolívia	-	✓	-	✓	-	-	-	✓
Brazil	-	✓	-	✓	-	✓	-	✓
Chile	-	✓	-	✓	-	NI	-	✓
Colombia	-	-	✓	✓	-	-	✓	✓
Costa Rica	-	✓	-	✓	-	-	✓	✓
Mexico	-	✓	-	✓	-	-	-	✓
Uruguay	-	-	-	-	-	-	-	-

-: not available; ✓: available; NI: not informed

**Table 3.** Clinical trials in TNBC were conducted in each country of LAC.

Country	Clinical trials^a^ (*n*)	Study phase *n* (%)								Status *n* (%)		Funder type^b^ *n* (%)			
		Early phase 1/Phase I		II		III		IV		Completed	Recruiting/active not recruiting	Industry		Others	
Mexico	12	0	0.0%	0	0.0%	12	100%	0	0.0%	1	11	12	92.3%	1	7,7%
Brazil	11	0	0.0%	1	9.1%	10	90.9%	0	0.0%	1	10	11	84.6%	2	15,4%
Argentina	11	0	0.0%	2	18.2%	9	81.8%	0	0.0%	2	9	11	91.7%	1	8,3%
Chile	8	1	12.5%	2	25.0%	5	62.5%	0	0.0%	1	7	8	100%	0	0,0%
Peru	7	0	0.0%	0	0.0%	7	100%	0	0.0%	1	6	7	87.5%	1	12,5%
Colombia	6	1	16.7%	2	33.3%	3	50.0%	0	0.0%	1	5	5	83.3%	1	16,7%
Costa Rica	2	0	0.0%	0	0.0%	2	100%	0	0.0%	1	1	2	100%	0	0,0%
Panama	1	0	0.0%	0	0.0%	1	100%	0	0.0%	0	1	1	100%	0	0,0%
Cuba	1	0	0.0%	0	0.0%	1	100%	0	0.0%	0	1	1	100%	0	0,0%
Total	19														

a Total number of clinical trials in the region

b Due to overlap or missing data, sums of the ‘Funder Type’ categories do not necessarily equal the total number of trials or 100% – source on Feb 23, 2023 (the National Library of Medicine 2023)
